# Improved detection of gallbladder perforation by contrast-enhanced ultrasound: two case reports

**DOI:** 10.3389/fmed.2024.1422708

**Published:** 2024-12-05

**Authors:** Yaxin Zhou, Lulu Yang

**Affiliations:** Department of Ultrasound, West China Hospital, Sichuan University, Chengdu, China

**Keywords:** gallbladder perforation, acute cholecystitis, contrast-enhanced ultrasound, diagnosis, case reports

## Abstract

Acute cholecystitis is a common acute abdominal disease, but the occurrence of secondary gallbladder perforation is relatively rare, and poses a serious threat to the lives of patients. The conventional methods for diagnosing acute cholecystitis are abdominal contrast-enhanced CT and ultrasound. However, these methods may have limitations in detecting gallbladder perforation, especially in cases where perforation size are small. In contrast, contrast-enhanced ultrasound (CEUS) has the distinct advantage of providing dynamic, real-time visualization of the gallbladder wall enhancement, thereby enabling a more confident diagnosis of gallbladder perforation. Here, we report two cases of acute cholecystitis initially diagnosed by abdominal contrast-enhanced CT, and secondary gallbladder perforation detected by further contrast-enhanced ultrasound. Both patients were finally treated in a timely manner. Our cases suggest that CEUS can be an important complementary method for diagnosing gallbladder perforation.

## Introduction

Acute cholecystitis is a frequently encountered acute abdominal disease characterized by the sudden onset of right upper quadrant pain, often accompanied by fever, nausea and vomiting. In severe cases, complications such as gangrene, perforation and abdominal sepsis may arise, posing a serious threat to the life of patients (1). Among these complications, gallbladder perforation is a relatively rare occurrence, with an incidence ranging from 2 to 12% in cases of acute cholecystitis (2, 3). Gallbladder perforation can be suspected clinically when patients have worsening abdominal pain, generalized tenderness and rebound tenderness. Also patient can easily go into sepsis and septic shock. Therefore, early and accurate diagnosis of gallbladder perforation is crucial for emergency department physicians to develop appropriate treatment plans, as the mortality rate of patients with gallbladder perforation can reach 12 to 16% (4).

Ultrasound is the initial diagnostic method for cholecystolithiasis, while CT scan is the preferred method for patients with acute abdomen, but studies have shown that the sensitivity and specificity of CT in diagnosing cholecystolithiasis are 80 and 93%, respectively, especially for non-calcified gallstones (5). Nevertheless, CT has a high diagnostic accuracy for acute cholecystolithiasis complicated infection, obstruction and other malignant diseases. Especially for patients with acute phlegmonous cholecystitis, ultrasound examination is limited because of pneumo-gallbladder at this time, while smaller gallbladder perforations can be challenging for CT imaging. Besides, the sensitivity and specificity of ultrasonography for diagnosing acute cholecystitis were 81 and 83%, respectively. However, in cases of gallbladder perforation, the ultrasound image can be complex due to variations in site of perforation, defect size, and disease progression; thus conventional ultrasound has limited preoperative diagnostic accuracy. As a novel ultrasonic examination technique, contrast-enhanced ultrasound (CEUS) can provide clinicians with important diagnostic information by accurately and clearly displaying the strengthening of the gallbladder wall and blood supply of space-occupying lesions in the gallbladder. In this study, we present two cases where accurate diagnosis and timely treatment were achieved through the use of CEUS for patients with gallbladder perforation. During CEUS, 2.4 mL of SonoVue ultrasound contrast agent (Bracco, Milan, Italy) was administered intravenously via the medial cubital vein and immediately followed by a 5 mL flush of 0.9% sodium chloride solution.

## Case presentations

**Figure 1:**
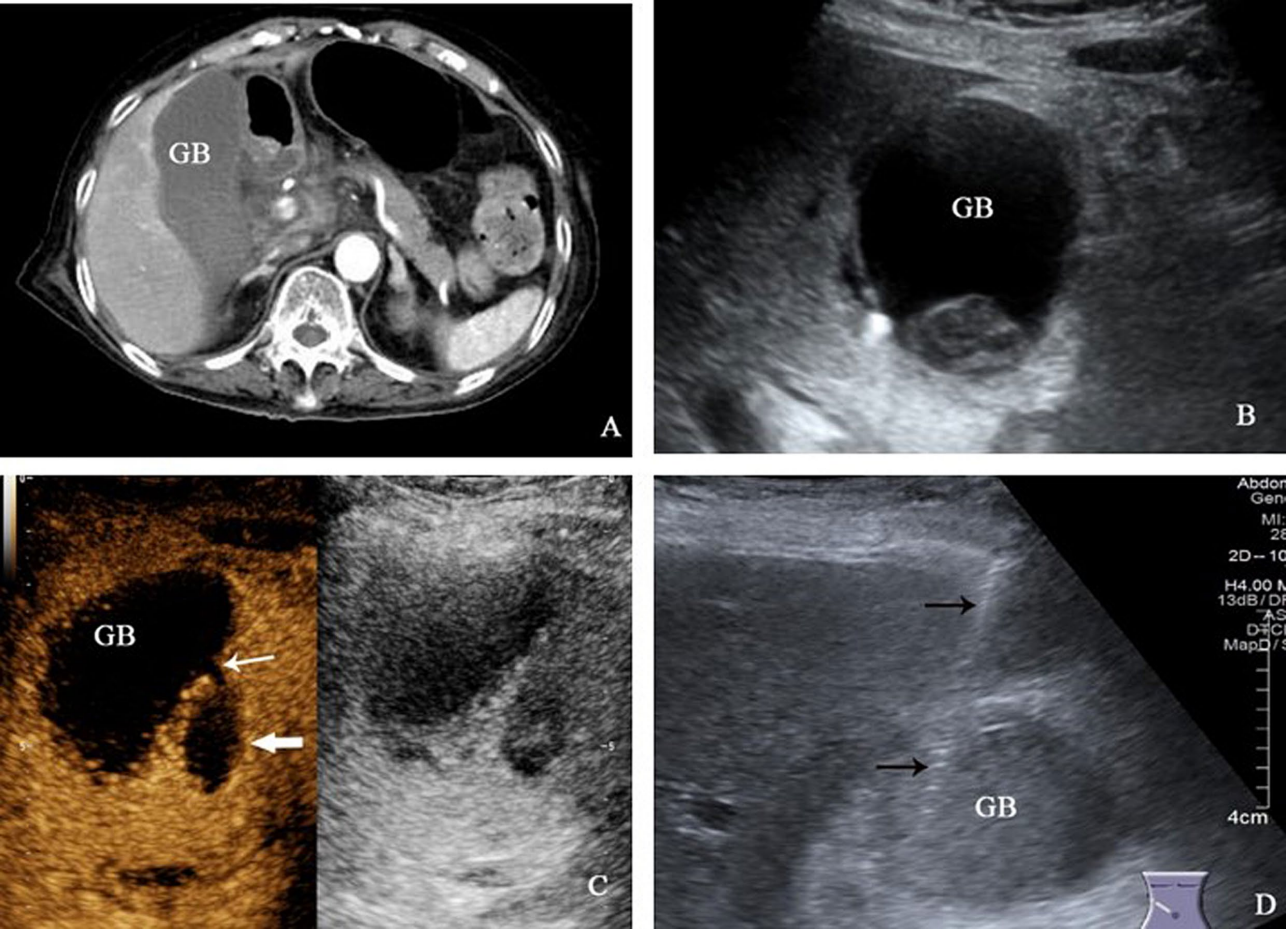
**(A)** The contrast-enhanced CT examination revealed a significant enlargement of the gallbladder, accompanied by the presence of low-density exudation shadow around it. **(B)** The gallbladder exhibited a transverse diameter of approximately 4.5 cm, with thickening of the gallbladder wall. Additionally, gallstones and viscous bile were observed within the cavity. **(C)** CEUS displayed a discontinuity in the left lateral wall of the gallbladder, with a local interruption of approximately 2 mm (thin white arrow), and an unenhanced area of 2.3 × 1.5 cm was visible beside the break (thick white arrow). **(D)** Percutaneous transhepatic gallbladder puncture under ultrasound guidance was performed, and the puncture needle was shown by thin black arrow.

Case 1 was an 82-year-old female patient who presented to the emergency department of our hospital with abdominal pain and vomiting for 1 day. The pain had worsened over the past 8 h. Physical examination revealed tenderness and rebound pain in the right upper abdomen, along with muscle tension, and tenderness in the lower abdomen. Laboratory tests showed the following results: red blood cell (RBC) count of 2.98 × 10^12^/L, hemoglobin (Hb) level of 102 g/L, white blood cell (WBC) count of 21.51 × 10^9^/L, neutrophils at 93.0%, lymphocytes at 4.1%, interleukin-6 level of 494 pg/mL, alanine aminotransferase (ALT) level of 54 IU/L, aspartate aminotransferase (AST) level of 123 IU/L, direct bilirubin (DBIL) level of 7.5 μmol/L, indirect bilirubin (IBIL) level of 9.6 μmol/L and albumin (ALB) level of 30.0 g/L. The contrast-enhanced CT suggested acute cholecystitis combined with localized peritonitis and gallbladder wall ischemia. Emergency abdominal ultrasound showed that the transverse diameter of the gallbladder was approximately 4.5 cm, with thickening of the gallbladder wall (0.7 cm at the thickest part) and gallstone and hypoechoic deposition in the cholecystic cavity. The diagnosis of acute cholecystitis was made based on the conventional ultrasound findings. Further CEUS showed inhomogeneous enhancement of the gallbladder wall, discontinuity of the left lateral wall (interrupted by approximately 2 mm), and a visible unenhanced area (2.3 × 1.5 cm) near the gallbladder at the site of the discontinuity, and no enhancement was observed in the gallbladder cavity, indicating acute cholecystitis with gallbladder perforation and perigallbladder abscess formation. Combined with the above examination results, the clinical diagnosis was acute cholecystitis with gallbladder perforation and localized peritonitis. Given the age of the patient and the presence of peritonitis, surgical intervention carries a high risk. Finally, the patient was treated with anti-infection medications [intravenous imipenem/cilastatin (500 mg every 8 h)] and underwent ultrasound-guided percutaneous transhepatic gallbladder drainage (Figure 1). After draining a large amount of black viscous bile, the clinical symptoms of the patient quickly improved, and recovered from the hospital 2 weeks later without undergoing cholecystectomy.

**Figure 2:**
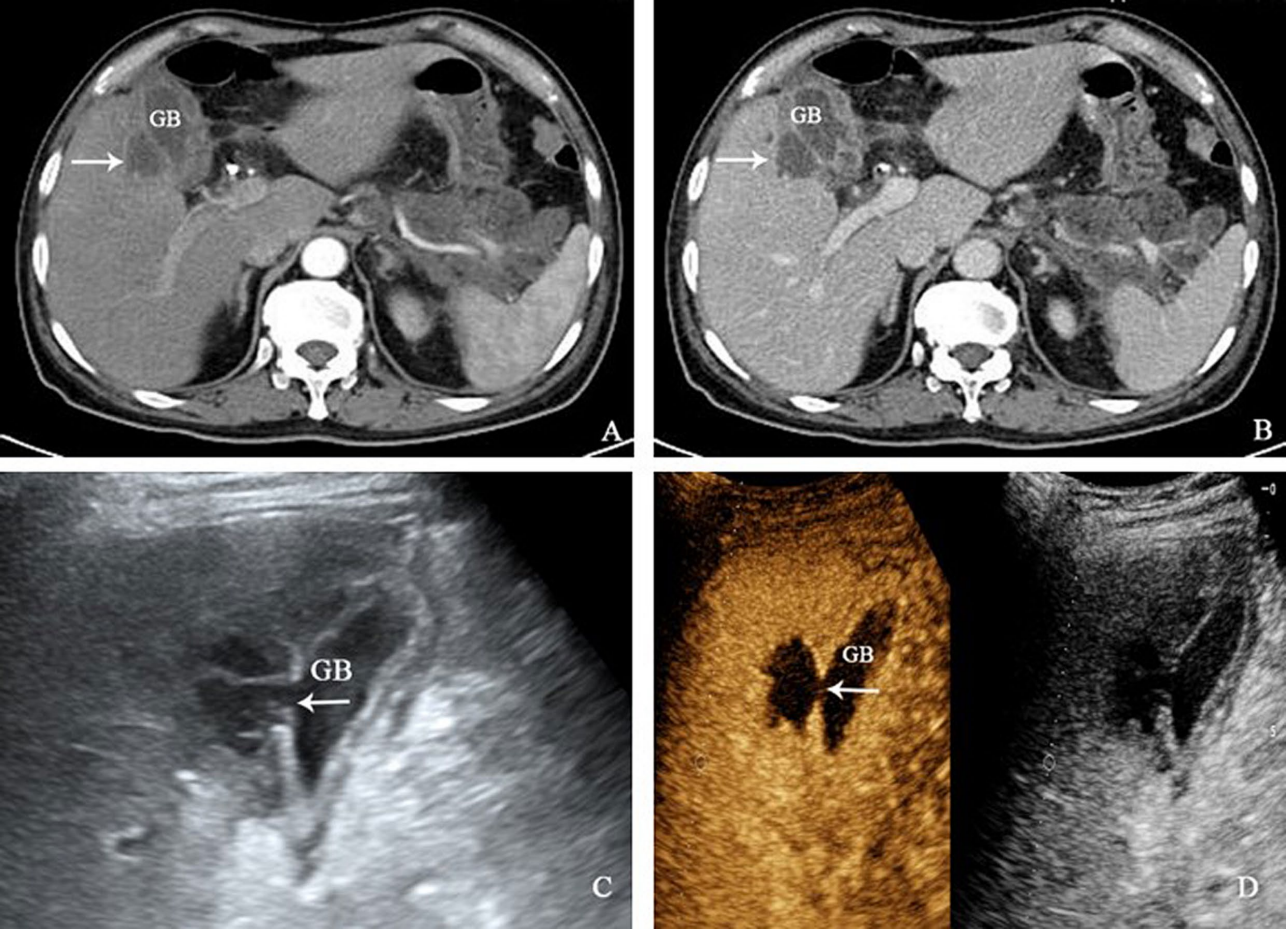
**(A,B)** The contrast-enhanced CT scan revealed thickening of the gallbladder wall, along with multiple cystic low-density shadows and enhanced edges within the wall. Furthermore, a 3.9 × 3.2 cm low-density mass with enhanced edges (white arrow) had formed in the right anterior lobe of the liver. **(C)** The right lateral wall of the gallbladder exhibited a discontinuity, with a local interruption in continuity of approximately 3.5 mm. Adjacent to the gallbladder wall, a mixed echo mass measuring about 4.2 × 3.4 cm was detected in the right anterior lobe of the liver. The margin between the liver mass and the gallbladder wall was indistinctly demarcated. **(D)** Following CEUS, the liver mass displayed uneven hyperenhancement during the arterial phase, along with obvious peripheral enhancement, hypoenhancement in the portal venous phase and delayed phase, and a non-enhanced area of 2.0 × 2.0 cm was observed within the liver mass.

Case 2 was a 67-year-old male patient who developed upper abdominal pain 1 month ago following ingestion of greasy food. Subsequent examinations confirmed acute pancreatitis, leading to hospitalization for treatment. Despite 1 month of medical intervention, his abdominal pain suddenly worsened. During the physical examination, tenderness and rebound pain were observed in the upper abdomen, mainly in the right upper quadrant. Laboratory tests revealed a RBC count of 3.41 × 10^12^/L, Hb level of 104 g/L, WBC count of 8.52 × 10^9^/L, neutrophilic granulocyte percentage of 76.0%, interleukin-6 level of 58.1 pg/mL, C-reactive protein level of 113 mg/L, ALT level of 63 IU/L, AST level of 60 IU/L, DBIL level of 6.1 μmol/L, IBIL level of 3.8 μmol/L and ALB level at 28.6 g/L. The xanthogranulomatous cholecystitis was suspected by contrast-enhanced CT, along with formation of a hepatic abscess in the adjacent liver tissue. The CEUS was subsequently performed. The conventional ultrasound revealed the gallbladder with a transverse diameter of 1.5 cm, exhibiting a discontinuous wall that was interrupted by approximately 3.5 mm in right lateral wall. Adjacent to the gallbladder wall, a mixed echoic mass with 4.2 × 3.4 cm was detected in the right anterior lobe of the liver, making it difficult to distinguish from the gallbladder wall itself. On CEUS, the liver mass exhibited heterogeneous hyperenhancement during the arterial phase, accompanied by pronounced peripheral enhancement, and hypoenhancement in the portal venous and late phase. Additionally, there was a non-enhanced area with 2.0 × 2.0 cm in size within the mass (Figure 2). Based on these findings, the clinical diagnosis was acute cholecystitis with gallbladder perforation and the subsequent formation of a liver abscess. Consequently, an emergency cholecystectomy was performed. Intraoperatively, it was observed that the gallbladder had enlarged to about 7.0 × 4.0 cm in size, and 0.3 cm in thickness, with the gallbladder wall exhibiting perforation. Several gallstones were also visible within the gallbladder, and a liver abscess, measuring approximately 4.0 cm, was found between the gallbladder wall and the liver. Fortunately, the patient had a successful recovery following surgery.

## Discussion

Gallbladder perforation is a serious complication of acute cholecystitis, with gallstone being a common but not exclusive cause, which is associated with high morbidity and mortality. Ultrasound is a preferred imaging modality for detecting gallbladder perforation and biliary pathology. However, conventional ultrasound examination often presents challenges in visualizing the site and extent of perforation due to blurring of the gallbladder wall caused by encapsulation from omentum and surrounding tissues (6). It has been reported that the ultrasonography display rate of gallbladder wall defect among patients with surgically confirmed gallbladder perforation is only 70%, while contrast-enhanced CT shows a higher rate at 78–100% (7). In recent years, CEUS has emerged as a valuable tool for evaluating gallbladder diseases, enabling identification of the nature of gallbladder lesions and assessment of the integrity of the gallbladder wall (8–10). During the early arterial phase of CEUS, rapid enhancement of the gallbladder walls results in hyperechoic intensity, enhancing contrast with surrounding tissue and improving visualization, which can be an alternative option for diagnose gallbladder perforation.

Due to the lack of specific clinical symptoms and laboratory indicators in patients with gallbladder perforation, clinical recognition can be challenging and sometimes delayed or ignored. In this study, the contrast-enhanced CT scans did not clearly indicate gallbladder perforation in these two patients, and conventional ultrasound also failed to detect gallbladder perforation in one case. However, CEUS clearly showed an interruption in the continuity of the gallbladder wall, allowing for an accurate assessment of the size of the defect and the presence of abscesses surrounding the gallbladder. These findings are crucial for clinicians to promptly develop appropriate treatment plans for gallbladder perforation. In one case, a percutaneous transhepatic gallbladder puncture drainage (PTGBD) procedure was performed under ultrasound guidance for an elderly patient accompanied by peritonitis. This intervention effectively alleviated the patient’s symptoms and prevented the worsening of her condition. It has been demonstrated in studies that PTGBD can lead to improved clinical outcomes, shorter hospital stays, reduced postoperative complications, and can even delay the need for a laparoscopic cholecystectomy (11, 12). The failure to perform cholecystectomy following cholecystostomy is correlated with adverse outcomes. Although the timing of cholecystectomy, whether early or delayed, has shown little disparity in outcomes (13). However, it should be noted that not all patients who undergo PTGBD require a cholecystectomy. Molavi et al. (14) found that 68.6% of patients with acute cholecystitis did not undergo delayed cholecystectomy after PTGBD. Similarly, Akasu et al. (15) suggested that, for elderly patients with acute cholecystitis and multiple comorbidities, cholecystectomy might not be necessary following PTGBD. The elderly patient with PTGBD in the study did not undergo cholecystectomy and was recovered from the hospital 2 weeks later.

Because bile leakage after gallbladder perforation can lead to inflammatory reactions in surrounding tissues of gallbladder, such as liver abscess or localized peritonitis, it is crucial to be highly alert to the possibility of gallbladder perforation in clinical practice when imaging findings indicate acute cholecystitis accompanied by peripheral encapsulated effusion or abscess. In some cases, when the fistula is small or the gallbladder atrophies due to recurring inflammation, the gallbladder tension is insufficient, and the gallbladder wall is irregular, CT images may be difficult to accurately judge the continuity of the gallbladder wall. The diameter of gallbladder perforation in the two cases in this study was small, ranging from 2.0 to 3.5 mm, which was difficult to detected through CT and conventional ultrasound, but indicated perigallbladder abscess. While the location and size of gallbladder perforation and the formation of peripheral abscess in both cases could be clearly displayed by CEUS. However, this study was limited by the number of cases, and a larger sample size is needed to further investigate the value of CEUS in detecting acute cholecystitis with gallbladder perforation.

In conclusion, gallbladder perforation is a rare yet potentially life-threatening condition that can pose challenges in clinical diagnosis. The utilization of CEUS may be able to assist in accurately diagnosing gallbladder perforation and providing an accurate evidence for prompt treatment of patients.

## Data Availability

The raw data supporting the conclusions of this article will be made available by the authors, without undue reservation.
